# OMA standalone: orthology inference among public and custom genomes and transcriptomes

**DOI:** 10.1101/gr.243212.118

**Published:** 2019-07

**Authors:** Adrian M. Altenhoff, Jeremy Levy, Magdalena Zarowiecki, Bartłomiej Tomiczek, Alex Warwick Vesztrocy, Daniel A. Dalquen, Steven Müller, Maximilian J. Telford, Natasha M. Glover, David Dylus, Christophe Dessimoz

**Affiliations:** 1 Swiss Institute of Bioinformatics, 1015 Lausanne, Switzerland;; 2 Department of Computer Science, ETH Zurich, 8092 Zurich, Switzerland;; 3 Centre for Mathematics and Physics in the Life Sciences and Experimental Biology (CoMPLEX), University College London, London WC1E 6BT, United Kingdom;; 4 Centre for Life's Origins and Evolution, Department of Genetics, Evolution & Environment, University College London, London WC1E 6BT, United Kingdom;; 5 Genomics England, Queen Mary University of London, London EC1M 6BQ, United Kingdom;; 6 Intercollegiate Faculty of Biotechnology, University of Gdansk and Medical University of Gdansk, 80-307 Gdansk, Poland;; 7 Department of Computational Biology, University of Lausanne, 1015 Lausanne, Switzerland;; 8 Center for Integrative Genomics, University of Lausanne, 1015 Lausanne, Switzerland;; 9 Department of Computer Science, University College London, London WC1E 6BT, United Kingdom

## Abstract

Genomes and transcriptomes are now typically sequenced by individual laboratories but analyzing them often remains challenging. One essential step in many analyses lies in identifying orthologs—corresponding genes across multiple species—but this is far from trivial. The Orthologous MAtrix (OMA) database is a leading resource for identifying orthologs among publicly available, complete genomes. Here, we describe the OMA pipeline available as a standalone program for Linux and Mac. When run on a cluster, it has native support for the LSF, SGE, PBS Pro, and Slurm job schedulers and can scale up to thousands of parallel processes. Another key feature of OMA standalone is that users can combine their own data with existing public data by exporting genomes and precomputed alignments from the OMA database, which currently contains over 2100 complete genomes. We compare OMA standalone to other methods in the context of phylogenetic tree inference, by inferring a phylogeny of Lophotrochozoa, a challenging clade within the protostomes. We also discuss other potential applications of OMA standalone, including identifying gene families having undergone duplications/losses in specific clades, and identifying potential drug targets in nonmodel organisms. OMA standalone is available under the permissive open source Mozilla Public License Version 2.0.

The sequencing revolution is yielding a flood of genomes and transcriptomes, with thousands already sequenced and many more underway (Pagani et al. 2012). A powerful way of characterizing newly sequenced genes is to compare them with evolutionarily related genes—in particular, with orthologs in other species (Dessimoz et al. 2012; Sonnhammer et al. 2014; Forslund et al. 2018). In this way, experimental knowledge from model organisms can be propagated to nonmodel organisms. Elucidation of orthology and paralogy relationships is also essential to reconstruct species trees, to better understand the mechanics of gene/genome evolution, to study adaptation, or to pinpoint the emergence of new gene functions (Gabaldón and Koonin 2013).

The importance of determining orthology has led to the development of many inference methods and associated databases (for review, see Altenhoff and Dessimoz 2012). Some of the best established orthology resources include eggNOG (Huerta-Cepas et al. 2016b), Ensembl Compara (Zerbino et al. 2018), InParanoid (Sonnhammer and Östlund 2015), MBGD (Uchiyama et al. 2012), OrthoDB (Zdobnov et al. 2017), OrthoMCL (Chen et al. 2006), PANTHER (Mi et al. 2017), PhylomeDB (Huerta-Cepas et al. 2014), and OMA (Altenhoff et al. 2018).

Key distinctive features of OMA are the high specificity of its inference pipeline (Altenhoff and Dessimoz 2009; Boeckmann et al. 2011; Linard et al. 2011; Afrasiabi et al. 2013), the feature-rich web and programmatic interfaces, large size and taxonomic breadth of its precomputed data (currently 2167 genomes), its regular update schedule of two releases per year, and its sustained development over the last 13 yr. The algorithms underlying the OMA pipeline have been described and validated in multiple publications (Dessimoz et al. 2005, 2006; Roth et al. 2008; Altenhoff et al. 2013; Train et al. 2017). The quality of OMA is corroborated by a recent community benchmarking study, which highlighted the high specificity of orthologs predicted by the OMA pipeline (Altenhoff et al. 2016).

With genome and transcriptome sequencing rapidly becoming a commodity, there is an increasing need to analyze custom user data. Here, we present OMA standalone, an open-access software implementation of the OMA pipeline for Linux and Mac (http://omabrowser.org/standalone). We first outline some of the key features of OMA standalone. In the second part, we demonstrate the usefulness of OMA standalone in the context of species tree inference, by comparing its performance with state-of-the-art alternatives on the challenging Lophotrochozoa phylogeny.

## Results

We first highlight the defining features of OMA standalone, then turn to the phylogeny of Lophotrochozoa, which we infer from orthologs inferred by OMA in comparison with alternative methods.

### OMA standalone software

OMA standalone takes as input the coding sequences of genomes or transcriptomes, in FASTA format. The recommended input type is amino acid sequences, but OMA also supports nucleotide sequences. With amino acid sequences, users can combine their own data with publicly available genomes from the OMA database, including precomputed all-against-all sequence comparisons (the first and computationally most intensive step), using the export function on the OMA website (http://omabrowser.org/export).

**Figure 1.**
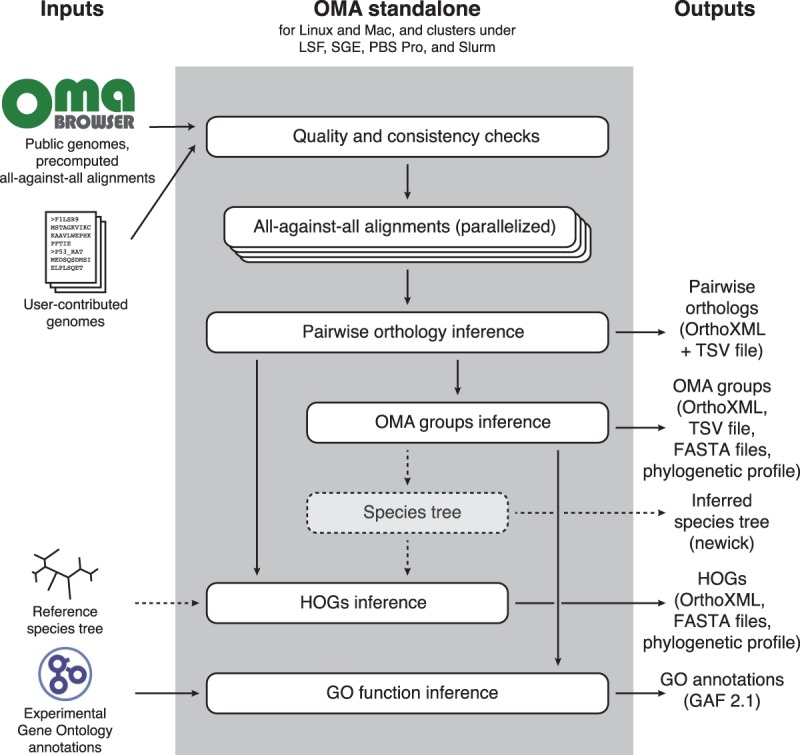
Conceptual overview of the OMA standalone software. Dotted arrows indicate alternative steps (reference species tree either specified as input or inferred from the data). The species tree inference step infers a distance tree but can be bypassed by supplying a reference tree.

OMA standalone produces several types of output (also summarized in Fig. 1):

1. Pairwise orthologs and their subtypes (one-to-one, one-to-many, many-to-one, many-to-many orthology). These orthologs are useful when comparing pairs of species or to identify orthologs to specific genes of interest.
2. OMA groups. These are sets of genes for which all pairs are inferred to be orthologous. These groups are inferred as cliques (fully connected subgraphs) of pairwise orthologs. These groups are not necessarily one-to-one orthologs, but, being inferred without assuming a species tree, they are particularly useful to identify marker genes for phylogenetic reconstruction.
3. Hierarchical orthologous groups (HOGs). These groups are defined for every internal node of the (rooted) species tree; each HOG contains the genes that are inferred to have descended from a common ancestral gene among the species attached to that internal node. Consider, for instance, gene *ADH1*, which duplicated within the primates (Carrigan et al. 2012): At the level of the last primate common ancestor, all genes that have descended from the ancestral *ADH1* belong to the same HOG. However, at the level of the common ancestor of all the great apes, because *ADH1* had at this point already duplicated into *ADH1A*, *ADH1B*, and *ADH1C*, these ancestral genes define three HOGs. A brief video tutorial on HOGs is available at https://youtu.be/5p5x5gxzhZA. The HOGs are stored in the standard OrthoXML format (Schmitt et al. 2011).
4. Gene Ontology annotations. OMA standalone annotates the input sequences with Gene Ontology annotations by propagating high-quality annotations across orthologs (Altenhoff et al. 2015). The annotations are provided in the standard GO Annotation File Format 2.1 (http://geneontology.org/docs/go-annotation-file-gaf-format-2.1).
5. Phylogenetic profiling. Orthology is also used to build phylogenetic profiling—patterns of presence and absence of genes across species (Pellegrini et al. 1999). We provide two forms of output: a binary matrix with species as rows and OMA groups as columns, indicating patterns of presence or absence of genes in each group; a count matrix with species as columns and HOGs as rows, indicating the number of genes in each deepest-level HOG (i.e., HOG defined at the broadest taxonomic level possible).
6. Species tree. Unless supplied with a (fully or partially resolved) reference species tree, OMA standalone computes a tree from the inferred OMA groups using the built-in distance tree procedure *MinSquareTree* in the programming environment *Darwin* (Gonnet et al. 2000). Note that, as with most tree inference methods, the rooting of the tree tends to be unreliable, so we encourage users to review and reroot the tree based on other information, if available.

**Figure 2.**
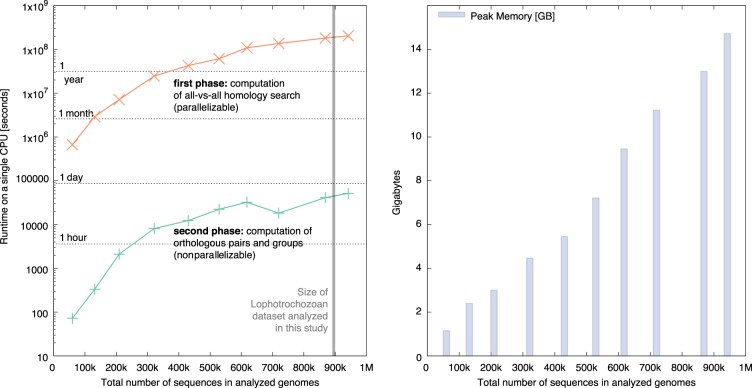
Resource measurements for various data sets of increasing sizes as total number of protein sequences. The data sets have been sampled from the public OMA Browser to maintain a constant composition of 20% fungi, 10% archaea, 10% plants, 20% metazoan, and 40% bacteria genomes. (*Left*) Runtime of the all-against-all phase (orange) on a single CPU, and the inference of the orthologous pairs and various groups (green). (*Right*) Peak memory usage of OMA standalone in gigabytes (GB).

OMA standalone supports parallel computation of the all-against-all sequence comparison phase. This phase, which computes Smith–Waterman (1981) alignments followed by pairwise maximum likelihood distance estimation for all significant pairs (Roth et al. 2008), is by far the most time-consuming step of the algorithm. To fully exploit parallelism, alignments are performed using single instruction multiple data (SIMD) instructions (Szalkowski et al. 2008) on multiple cores. OMA standalone natively supports common cluster schedulers—LSF, SGE, PBS, and Slurm—and has been successfully run with several thousand jobs in parallel. Figure 2 shows typical runtimes and memory usage for data sets of various sizes.

### Application: the phylogenetic relationships within Lophotrochozoa

Resolving the relationships of ancient lineages is a major challenge for molecular phylogenetics. Although some aspects of the phylogeny of the major animal clades are well-resolved, the relative positions of the deeper lying clades are often disputed. The construction of large phylogenomic supermatrices has been the method of choice for resolving the deepest nodes in the tree of life (Dunn et al. 2008; Hejnol et al. 2009; Fernández et al. 2014; Egger et al. 2015).

Fundamental to the analyses of phylogenetic relationships is the use of sequences that have descended from a single common gene in their last common ancestor, that is, orthologous sequences. Ensuring that we correctly infer orthologs is therefore vital if we are to reconstruct difficult to resolve phylogenies. The limitations of automated orthology and paralogy prediction methods with regard to phylogenetic analysis have previously been highlighted (Philippe et al. 2011b); simplistic orthology inference methods may miss orthologs (Dalquen and Dessimoz 2013) or erroneously identify paralogous pairs of genes as orthologs as a result of differential gene losses (Dessimoz et al. 2006).

One notoriously difficult to resolve phylogeny is that of Lophotrochozoa (Kocot 2016), a clade of animals positioned sister to Ecdysozoa, within the protostomes, and which, for instance, includes segmented worms and molluscs. Lophotrochozoa contains about 10 different phyla, each of which is clearly monophyletic, but the relationships among these phyla are far from clear, with many different topologies having been supported by different analyses. The inference is that the phyla are likely to have emerged in an ancient and rapid radiation resulting in weak phylogenetic signal for interphylum relationships. These circumstances make the solving of this problem particularly difficult and mean that the use of accurately identified orthologs is particularly relevant.

We used OMA standalone to identify orthologous marker genes among the proteomes of 19 lophotrochozoans and, as outgroups, four deuterostomes, four ecdysozoans, and three nonbilaterians, totaling 894,528 input sequences (see Methods). As a basis of comparison, we also repeated the analysis using orthology inference pipelines, on the same data set, based on OrthoMCL (Li et al. 2003), BUSCO (Simão et al. 2015), HaMStR (Ebersberger et al. 2009), and OrthoFinder (Emms and Kelly 2015). Like OMA, these methods do not require prior specification of a species tree, are available as standalone programs, and have all been used in phylogenetic analyses previously. Species trees were then constructed using these orthologs with both maximum likelihood and Bayesian tree reconstruction packages, IQ-TREE (Nguyen et al. 2015) and PhyloBayes (Lartillot et al. 2013), on the resultant supermatrices. In terms of computational cost, OMA is by far the most costly of the orthology methods tested, due to its reliance on full Smith–Waterman (1981) alignments and evolutionary distance in the all-against-all phase (∼85 k CPU hours). By comparison, OrthoMCL and OrthoFinder, which rely on BLAST for all-against-all comparisons, are much faster (∼2 k CPU hours). Finally, BUSCO (11 CPU hours) and HaMStR (230 CPU hours) are the fastest, owing to their reliance on predefined hidden Markov models of the orthologous markers.

**Figure 3.**
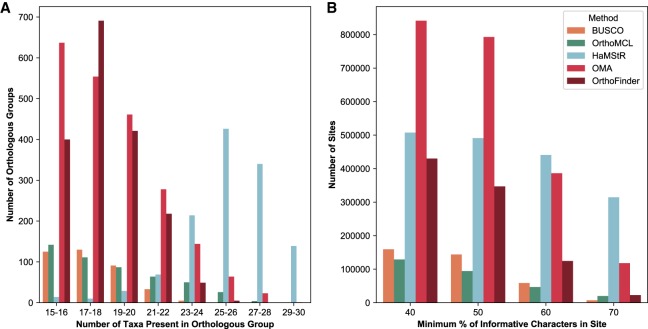
Comparison of amount of orthologous data inferred by the different pipelines. (*A*) OMA and OrthoFinder infer more orthologous groups than other methods, whereas the groups inferred by HaMStR are considerably larger on average than for the other methods. (*B*) The resulting supermatrix has most sites for OMA, whether the minimum site occupancy threshold is 40% or 50%, and most sites for HaMStR at the 60% cutoff (used for phylogenomic reconstruction) and 70% cutoff.

We first consider the amount of orthology information recovered by the various methods. OMA inferred 2162 orthologous groups containing 15 or more species (Fig. 3A). By comparison, the HaMStR pipeline inferred 1241 orthologous groups, the OrthoMCL pipeline inferred 484 orthologous groups, BUSCO inferred 384 orthologous groups, and OrthoFinder yielded 1784 groups. Although OMA identifies more orthologous genes than other methods, it infers fewer larger groups than HaMStR and produces a less dense data matrix (Supplemental Fig. S1). This difference in group size distribution is likely to be the result of different trade-offs in terms of precision (proportion of predicted orthologs that are correct) and recall (proportion of true orthologs that are correctly predicted). These trade-offs have been observed in multiple benchmarking studies (e.g., Boeckmann et al. 2011; Altenhoff et al. 2016). Indeed, the OMA algorithm is known for having higher precision but lower recall than most other methods (Altenhoff et al. 2016).

A priori, the effect of the number of orthologous groups and completeness on tree inference is not obvious. The effect of missing data in even large supermatrices has been shown to have a detrimental effect on the quality of trees inferred from them (Roure et al. 2013). Other studies have shown that more complete supermatrices do not necessarily yield better results (Fernández et al. 2016). The latter study found that when using a stringent minimal site completeness cut-off, resulting in fewer sites, phylogenetic inference was in disagreement with established classifications of taxa.

If we consider both the number of sites above a minimum occupancy rate threshold (i.e., minimum proportion of informative characters in each site), OMA standalone yields the largest data matrices (i.e., the most alignment columns) with at least 40% or 50% occupancy, while HaMStR yields the largest data matrices for 60% and 70% (Fig. 3B).

Using the aligned sets of orthologs identified in the previous step, we reconstructed species trees using Maximum Likelihood (IQ-TREE [Nguyen et al. 2015], a model selected with ModelFinder [Kalyaanamoorthy et al. 2017]), and Bayesian analysis (PhyloBayes, CAT + GTR + G4 [Lartillot et al. 2013]) on supermatrices that had been filtered to include only alignment columns with at least 60% site occupancy. In the rest of our analyses, we chose to infer trees from matrices with a minimum occupancy rate of 60%, for pragmatic reasons: With higher thresholds, some methods recover too few sites (e.g., BUSCO yields 7135 positions only if we require at least 70% occupancy). With a lower cutoff, the increase in data matrix size renders Bayesian tree inference analyses prohibitively costly.

**Figure 4.**
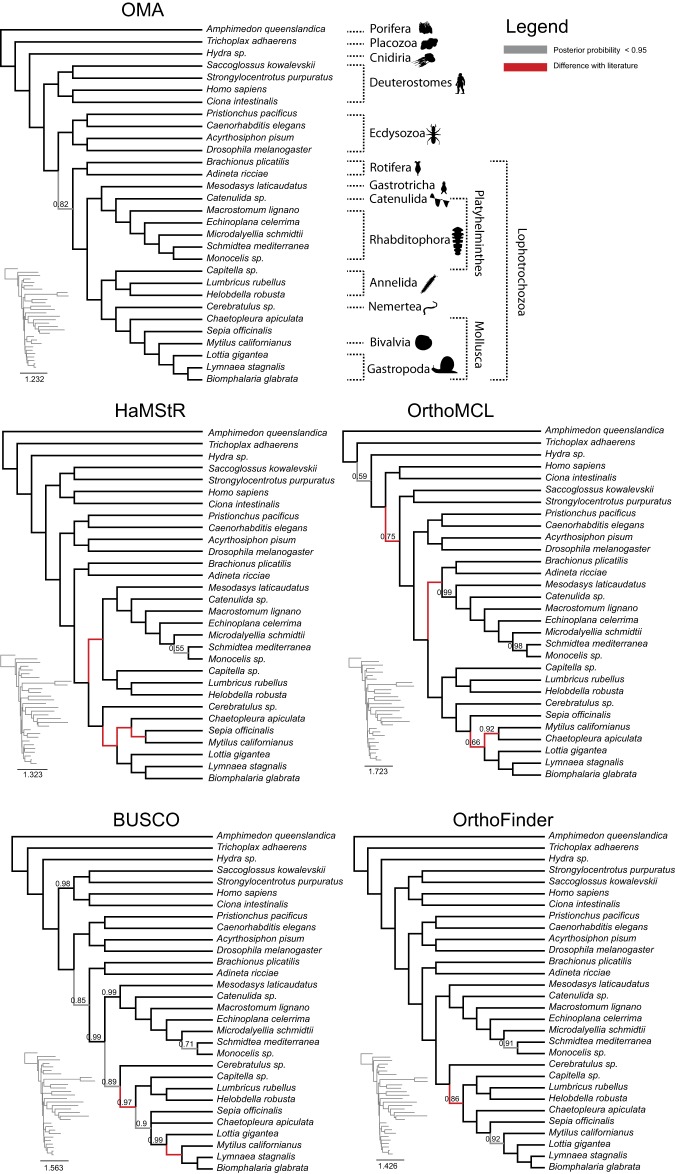
Comparison of trees obtained using PhyloBayes with the CAT-GTR-G4 model from the different orthology methods. OMA tree is in congruence with published results (see main text). Branches that are at odds with the literature are in red; otherwise they are displayed in gray (posterior probability < 0.95) or else in black. Only posterior probabilities below one are displayed. Please note that the PhyloBayes tree computed from HaMStR data did not converge after 900,000 CPU hours and thus should be interpreted with caution.

With OMA, both the Bayesian tree (using PhyloBayes; Fig. 4) and the ML tree (using IQ-TREE; Supplemental Fig. S2) had high branch support values. The Bayesian tree had branch posterior probabilities of 1 across the tree apart from the Lophotrochozoa clade, with a posterior probability of 0.82. The ML tree had bootstrap support of 100 for all but eight of 27 branches. Deuterostomes were recovered with full bootstrap support, while Lophotrochozoa, with the exception of Rotifera, were recovered with bootstrap support of 92.

The OMA tree inferred using the ML inference method found that the Rotifera (*Adineta ricciae, Brachionus plicatilis*) are grouped with the Nematoda (*Caenorhabditis elegans*, *Pristionchus pacificus*), as part of the ecdysozoans. This is in disagreement with the current consensus (Giribet and Edgecombe 2017). In contrast, the tree constructed using Bayesian inference found the Rotifera to be sister to the rest of the lophotrochozoans, in agreement with recent studies (Philippe et al. 2011a; Egger et al. 2015). The discrepancy in the ML tree is likely due to the long branched Rotifera being attracted to the long branched Nematoda—a problem to which PhyloBayes under the CAT model has been previously shown to be more robust (Lartillot et al. 2013).

Both the ML and Bayesian trees found the rest of the lophotrochozoans to consist of two monophyletic groups. The first group comprises the Gastrotricha (*Mesodasys laticaudatus*) and the Platyhelminthes (flatworms). This relationship is consistent with recent studies (Dunn et al. 2008; Edgecombe et al. 2011; Struck et al. 2014; Laumer et al. 2015). Because of their seemingly simple morphology, with characteristics such as having no body cavity, no respiratory organs, and having only a single opening for both the intake of nutrients and excretion of waste, they were originally thought to be among the most basally branching Bilateria, until molecular studies on 18S rDNA sequence data was carried out, placing them within the protostomes (Baguñà and Riutort 2004). Authors now divide the Platyhelminthes into the Catenulida, with currently no known synapomorphies (i.e., no shared distinctive character), and the Rhabditophora, which has uniting characteristics such as the presence of lamellated rhabdites, a common structure of the epidermis (Egger et al. 2015; Laumer et al. 2015). Our ML and Bayesian trees corroborated this and found the Catenulida (*Catenulida* sp.) to be sister to Rhabditophora (*Macrostomum lignano, Echinoplana celerrima, Microdalyellia schmidtii, Monocelis* sp., *Schmidtea mediterranea*).

Within the Rhabditophora, the most basal branches of the OMA-inferred trees are those of the Macrostomorpha (*Macrostomum lignano*), followed by the Polycladida (*Echinoplana celerrima*), also in agreement with recent studies (Egger et al. 2015; Laumer et al. 2015). We also inferred the Rhabdocoela (*Microdalyellia schmidti*) to be the most basally branching, followed by the Proseriata (*Monocelis sp.*) and Acentrosomata (*Schmidtea mediterranea*). This too is in agreement with recently published phylogenies (Egger et al. 2015; Laumer et al. 2015).

The second monophyletic group found within the rest of Lophotrochozoa contains the Annelida (*Lumbricus rubellus*, *Helobdella robusta*, *Capitella* sp.), segmented worms, the Mollusca (*Biomphalaria glabrata*, *Lymnaea stagnalis*, *Lottia gigantea*, *Mytilus californianus*, *Sepia officinalis*, *Chaetopleura apiculata*), the largest marine phylum, and Nemertea (*Cerebratulus* sp.), also known as ribbon worms or proboscis worms, to form the Trochozoa (Dunn et al. 2014). However, there is disagreement on the positioning of these clades within the group (Dunn et al. 2008; Struck and Fisse 2008; Struck et al. 2014; Laumer et al. 2015). Both tree reconstruction methods find the Gastropoda (*Lottia gigantea*, *Lymnaea stagnalis*, *Biomphalaria glabrata*) to be sister to the Bivalvia (*Mytilus californianus*). Both methods also found the Annelida to be sister to (Mollusca + Nemertea), with high support (posterior probability of 1 and bootstrap of 96).

In contrast, on this lophotrochozoan data set, trees obtained from other orthology pipelines had more unresolved nodes and/or more discrepancies with the literature (Fig. 4; Supplemental Table S1; Dunn et al. 2008; Kocot et al. 2011; Egger et al. 2015; Laumer et al. 2015; Telford et al. 2015; Kocot et al. 2017).

The BUSCO Bayesian tree had slightly less support throughout than the OMA tree, although it only had one branch with support of less than pp = 0.80. The relationship between the Proseriata, Rhabdocoela, and the Acentrosomata agrees with the OMA Bayesian tree, as does the relationship between the Gastrotricha and the Platyhelminthes. However, the BUSCO tree indicates Gastropoda to be paraphyletic with high support (pp = 0.99), with *Lottia gigantea* more basally branching to the Bivalvia and the rest of the Gastropoda. This is in contrast to both the OMA tree and other studies (Dunn et al. 2008; Struck et al. 2014). The BUSCO tree found the Nemertea as sister to (Annelida + Mollusca), with a support value of pp = 0.89. This is in disagreement with the current consensus and the OMA tree (Dunn et al. 2008; Struck et al. 2014; Laumer et al. 2015).

The HaMStR tree had high support throughout but differed markedly from the OMA tree. The HaMStR method placed *Sepia officinalis, Mytilus californianus*, and *Chaetopleura apiculata* in a clade together, sister to the Gastropoda. This is in disagreement with Kocot et al. (2011) and the OMA trees, which place the Polyplacophora (*Chaetopleura apiculata*) to be the most basally branching, followed by the Cephalopoda (*Sepia officinalis*), with the Bivalvia sister to the Gastropoda. The Bayesian tree also fails to recover Trochozoa, placing the Annelida with the (Platyhelminthes + Gastrotricha), as opposed to full support found in the OMA tree. One caveat with the Bayesian HaMStR tree is that the tree reported is unconverged (even after 22,230 iterations); thus, we cannot rule out that some of these differences might ultimately disappear. However, the ML tree also shows substantial disagreement with the OMA tree and the literature (Supplemental Table S1).

The OrthoMCL trees had the most issues, with the lowest support values. Deuterostomes, comprising a well-established relationship between the chordates and the Ambulacraria (Philippe et al. 2011a), are paraphyletic in the Phylobayes tree, which places chordates (*Ciona intestinalis, Homo sapiens*) more basally branching than the Ambulacraria (*Strongylocentrotus purpuratus, Saccoglossus kowalevskii*), with the latter sister to the Protostomes with pp = 0.75. Rotifera were incorrectly placed as sister to (Gastrotricha + Platyhelminthes) with full support. This is in disagreement with both the OMA tree and recent studies. The tree was able to correctly infer the (Mollusca + Nemertea) relationship with full support. Within the Mollusca, in contrast to the OMA tree, the Bayesian tree inferred *Sepia officinalis* to be the most basally branching, with *Chaetopleura apiculata* and *Mytilus californianus* forming a clade sister to the rest of the Mollusca. However, this has low support with pp = 0.66 for the Bayesian tree.

The OrthoFinder Bayesian tree was less supported than the OMA tree, with three values below pp = 1. The Nemertea were found to be sister to (Annelida + Mollusca), in contrast to the OMA Bayesian tree. The ML tree was also weakly supported, with nine branches with less than full support and six below bs = 80. The Rotifera were found to be sister to Platyhelminthes, as part of a clade with the Gastrotricha. This is in disagreement with recent analysis, which places them as sister to the rest of the lophotrochozoans. The phylogeny of the Mollusca differed from the OMA tree, with *Chaetopleura apiculata* and *Sepia officinalis* inferred as sister to one another, with bs = 44, which were in turn sister to (Bivalvia + Gastropoda).

**Figure 5.**
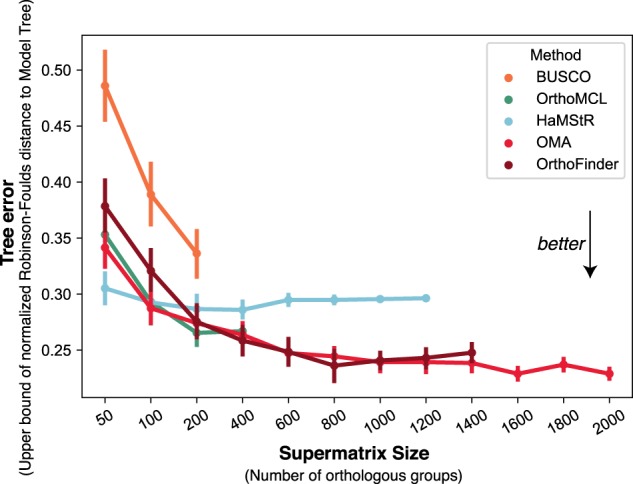
Accuracy of trees reconstructed with varying number of orthologous groups, on the lophotrochozoan data set, using IQ-TREE with a WAG + I model. Each point is obtained by averaging over results obtained from 50 random group subsets of varying size, drawn without replacement. Even if all methods are downsampled to have the same number of groups, trees obtained from OMA are consistently among the most accurate ones (measured in terms of the Robinson-Foulds distance to a partially resolved reference tree) (see Methods). Error bars depict one standard error on each side.

The different data matrices used to build phylogenies span an almost 10-fold difference in terms of informative sites. To better understand the potential impact of these differences, we sought to compare the quality of trees obtained from matrices subsampled to similar sizes, still on the lophotrochozoan data set. From each of the sets of orthologous groups produced by each method, a number of orthologous groups were selected at random, without replacement, but nevertheless ensuring that every species was represented at least once. For this analysis, which required the reconstruction of many species trees, we used IQ-TREE under the WAG + I model—which we found to be a reasonable trade-off between speed and accuracy. To gauge the accuracy of the resulting trees, we compared them with a partially resolved reference tree derived from the literature (see Supplemental Table S1). We observed that the lower accuracy of trees reconstructed from BUSCO and OrthoMCL is not solely due to the lower number of orthologous groups they infer: The resulting trees were less accurate even when we considered the same number of groups for all methods (Fig. 5). More generally, the analysis shows the merit of including more orthologous groups, as for most methods this leads to an increase in tree accuracy.

## Discussion

OMA standalone enables researchers to infer high-quality orthologs among genomes or transcriptomes, on public and in-house data. It runs on a wide range of hardware, from a single computer to large clusters with thousands of parallel processes.

A key application of OMA standalone lies in the identification of genome-wide orthologous marker sequences to infer difficult species phylogenies. On the lophotrochozoan data set, compared with other approaches, OMA yielded more orthologous information for phylogenetic species tree inference and resulted in better resolved trees, which are also more consistent with the existing literature. BUSCO finds orthologs by comparing sequence data to a predefined set of genes present in at least 90% of the species in a given data set. This relies on preexisting knowledge of orthology relationships in a set of reference species, in this case, the species present in the Metazoa data set. Therefore, the number of orthologous groups is limited to 843. Similarly, HaMStR relies on predefined core orthologs, which in our case were obtained from a high-profile previous study of the Annelida phylogeny (Weigert et al. 2014b). One advantage of such predefined sets is that phylogenetically uninformative or misleading genes might have already been excluded. The downside is that the number of orthologous groups is limited to 1253 orthologous groups. OMA is advantageous in this regard because it infers orthologs across potentially all protein sequences. This may in part explain why OMA standalone has been adopted in other phylogenomic studies, such as for centipedes (Fernández et al. 2014), arachnids (Sharma et al. 2014; Fernández and Giribet 2015), assassin flies (Dikow et al. 2017), scorpions (Sharma et al. 2015), spiders (Garrison et al. 2016), flatworms (Egger et al. 2015; Laumer et al. 2015), tapeworms (Tsai et al. 2013), Spiralian (Marlétaz et al. 2019), or Archaea (Williams et al. 2017).

Our comparison also has methodological implications for phylogenomic studies. These studies are typically greatly concerned about the impact of the evolutionary model on tree inference (e.g., Song et al. 2012), as well as that of taxon sampling (e.g., Dunn et al. 2008), but the impact of orthology inference methods has not nearly been as commonly investigated. Our comparison of orthology methods on the lophotrochozoan data set highlights the considerable impact orthology inference can have on phylogenetic tree inference. Thus, a more systematic investigation of the impact of orthology inference on phylogenetic tree inference may be required to resolve the most vexing phylogenetic questions, such as that of the ctenophore placement (Pisani et al. 2015, 2016; Whelan et al. 2015, 2017; Halanych et al. 2016; Feuda et al. 2017).

One drawback of the current OMA algorithm is its high computational cost compared to the other methods. It would be possible to replace the costly Smith–Waterman alignments by fast heuristics such as DIAMOND (Buchfink et al. 2015) or MMSeq2 (Steinegger and Söding 2017). However, currently about half of the time spent in the all-against-all phase is to compute pairwise evolutionary distances, which would still be needed—thus fast heuristics would only provide a 2× speed-up to the OMA pipeline at best. Instead, we see potential in avoiding the computation of some pairs altogether by exploiting the transitivity property of homology (Wittwer et al. 2014).

The comparative analysis has some limitations. First, the taxon sampling is far from optimal, with several clades, such as the rotifers, suffering from long branches. Since we started this study, more lophotrochozoan genomes have become available; their inclusion would likely improve the resolution of the trees. Second, while running and comparing five orthology inference methods on a data set of nearly 900,000 sequences already represents a major undertaking, other orthology methods would be interesting as well—in particular, tree-based approaches that require no prior species tree knowledge (Yang and Smith 2014; Huerta-Cepas et al. 2016a).

Beyond species tree inference, OMA can also be used to pinpoint the emergence of gene families in evolution, an approach that is sometimes referred to as phylostratigraphy (Domazet-Lošo et al. 2007). Conventional approaches work by considering all the genes annotated in a species of reference and performing BLAST searches against increasingly distant sets of taxa. The point at which no homolog can be found is inferred to immediately precede the emergence of the gene. However, such an approach does not differentiate between orthologs and paralogs and thus has a limited resolution in terms of subfamilies. Alternatively, it is possible to extract more fine-grained information from reconciled gene trees—i.e., gene trees with internal nodes labeled as speciation or duplication nodes (Vilella et al. 2008; e.g., Huerta-Cepas et al. 2014)—but this is computationally demanding and there is a lack of tools to perform such analyses on custom data.

By inferring high-quality hierarchical orthologous groups, OMA standalone provides a way to map gene emergence, gene duplication, and gene loss onto species phylogenies. For instance, OMA standalone has been used to contrast gene families that have expanded and contracted in the common ancestors of echolocating and nonecholocating bats. The emergence of echolocation coincides with a decrease in chemosensory genes, while secondary loss of echolocation coincides with an increase in chemosensory genes (Tsagkogeorga et al. 2017). The hierarchical orthologous groups inferred by OMA standalone can be further analyzed using the *iHam* visualization tool and the *pyHam* Python library (Train et al. 2018).

Orthology is also key to integrating biological knowledge among model and nonmodel species. Particularly when dealing with deep timescales, it can be challenging to identify genes with or without orthologous counterparts. By reconstructing fine-grained orthology between mice and protostomes, OMA standalone could identify new drug targets for neglected tropical diseases (Tsai et al. 2013). With such diseases, which disproportionately affect poorer people, it can be challenging to develop new medicines. To accelerate drug development in such cases, drug repurposing has been suggested whereby an already existing and approved medicine, or a well-researched lead, is used to combat neglected tropical diseases (Ekins et al. 2011). As a first-pass bioinformatic identification of drug targets in four newly sequenced tapeworm genomes, OMA standalone was used to identify orthologs of known human drug targets (Tsai et al. 2013): Human genes targeted by drugs were retrieved from various databases, and their orthologs in tapeworms were inferred using OMA standalone. To identify targets likely to be essential across animals, orthologs present in both mice and nematodes were also identified: If both mice and nematode orthologs had knock-out phenotypes, we inferred that the orthologous group was essential across animals. Together with other indicators, such as gene expression data, we were able to rank every gene in these largely unexplored genomes for their suitability as a drug target and associate lead compounds to them. As drugs could exhibit off-target effects on paralogs, the analysis focused on orthologs, which tend to be functionally more conserved (e.g., Altenhoff et al. 2012). The importance of investigating orthologs was illustrated by the drug Praziquantel, which is efficient against adult tapeworms but not against the more dangerous larval form (Nogi et al. 2009). Praziquantel targets one particular voltage-gated calcium channel subunit. Using OMA standalone, we could identify the precise subunit ortholog in tapeworms and show that it is not expressed in the larval form, thereby providing a plausible explanation for the drug's low efficacy.

To conclude, orthology inference is a key step in integrating biological knowledge across multiple species. OMA standalone is a versatile orthology inference software with a proven track record. Contrary to some of the orthology methods considered in this study, it was designed from the onset with species tree inference in mind, though it has since been applied for a broad range of other applications. The OMA standalone software implementation has been continuously improved and maintained over the past 5 yr, undergoing two major and 25 minor releases—in the course of which a considerable number of bugs were identified and fixed (https://omabrowser.org/standalone/release_notes.txt). We intend to keep developing and maintaining it. For support enquiries or bug reporting, we encourage users to use the biostars.org forum using the keyword “oma” (https://www.biostars.org).

## Methods

### OMA standalone

The list of all parameters of OMA standalone, their meaning, and default values is provided in Table 1.

**Table 1.**
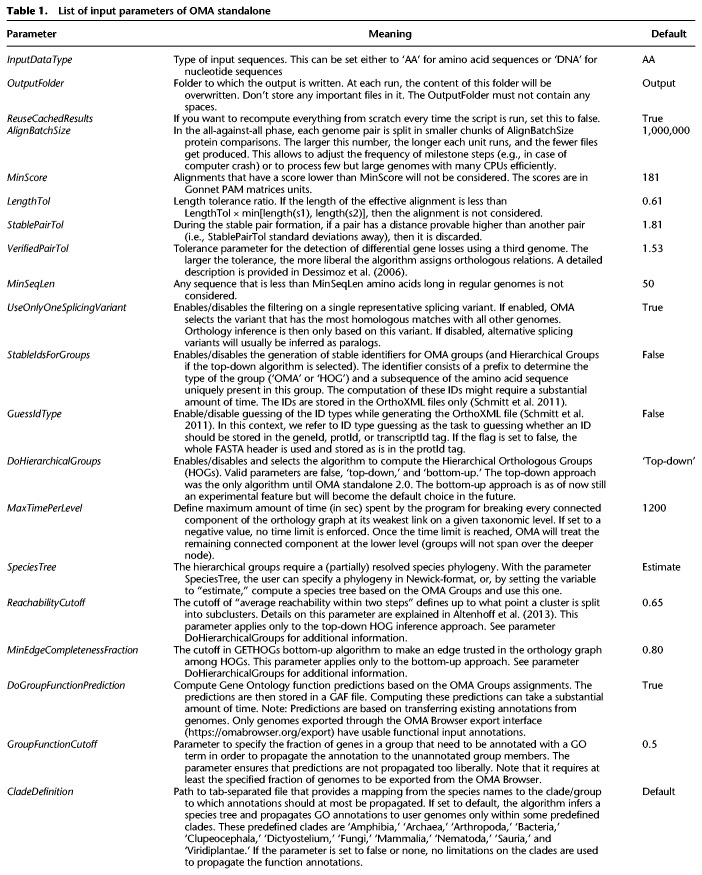
List of input parameters of OMA standalone

### Large-scale species phylogenetic reconstruction: Lophotrochozoa

#### Transcriptome assembly and peptide prediction

We used transcriptomes from seven Lophotrochozoa species published in Egger et al. (2015): *Mesodasys laticaudatus* (Gastrotricha), *Catenulida* sp., *Macrostomum ligano*, *Echinoplana celerrima*, *Microdalyellia schmidtii*, *Monocelis* sp. (Platyhelminthes), and *Cerebratulus* sp. (Nemertea). In addition, 12 sets of genomic and transcriptomic protein predictions from *Saccoglossus kowalevskii*, *Brachionus plicatilis*, *Adineta ricciae*, *Schmidtea mediterranea*, *Lumbricus rubellus*, *Chaetopleura apiculata*, *Sepia officinalis*, *Mytilus californianus*, *Biomphalaria glabrata*, *Lymnaea stagnalis*, *Hydra magnipapillata*, and *Amphimedon queenslandica* were downloaded from the NCBI RefSeq repository (ftp://ftp.ncbi.nlm.nih.gov/refseq/).

Quality assessment of sequencing reads was carried out with FastQC (https://www.bioinformatics.babraham.ac.uk/projects/fastqc/). Subsequent to this, it was determined, using PRINSEQ lite (Schmieder and Edwards 2011), that the first 12 nucleotides should be trimmed off the 100-bp reads. The assembly of the trimmed paired reads was done using Trinity v20130225 (Haas et al. 2013), with the flag ‘--min_kmer_cov 2’, with default parameters. Open reading frames (ORFs) were predicted using TransDecoder (Haas et al. 2013). All ORFs greater than 100 amino acids were retained. Redundant sequences with higher than 97% identity at the amino acid level were removed by clustering with CD-HIT (Fu et al. 2012).

In order to detect the presence of cross contaminations between the various libraries run on the same flow cell, we used the CroCo package (Simion et al. 2018). This identified any assembled transcripts with fewer than four read matches, which were subsequently discarded. Furthermore, this also discarded all transcripts in which the number of reads, from the intended species matching the transcript, was not at least five times greater than the number of matches to the transcript, from reads from any of the other potentially contaminating species.

Additionally, 11 precomputed proteomes for *Homo sapiens*, *Strongylocentrotus purpuratus*, *Ciona intestinalis*, *Trichoplax adhaerens*, *Pristionchus pacificus*, *Caenorhabditis elegans*, *Drosophila melanogaster*, *Acyrthosiphon pisum*, *Capitella* sp., *Helobdella robusta*, and *Lottia gigantea* were downloaded from the OMA database website. The combined set of 30 nonredundant protein sets contained 19 lophotrochozoans, four deuterostomes, four ecdysozoans, and proteomes from three nonbilaterian animals.

#### Orthology inference

For the HaMStR analysis, putative orthologs were determined for each species using HaMStR v13.1 (Ebersberger et al. 2009) using the Lophotrochozoa core ortholog reference data set (Weigert et al. 2014a,b) as required by the HaMStR tool, with default parameters. HaMStR was run with the “-representative” option to pick at most one sequence per species, with all other parameters as default.

Orthologous groups were inferred by running BUSCO v1.22 (Simão et al. 2015) on the Metazoa data set found at https://busco.ezlab.org/v1/. We created orthologous groups made up of the protein sequences which BUSCO deemed to have had complete matches with their own highly conserved genes. At most, one species containing multiple sequences was allowed per group. There was only a single occurrence of a group containing more than one species with multiple sequences. In this case, we retained only the longest sequence.

The set of 30 proteomes were first filtered to remove low-quality protein sequences using the OrthoMCL script “orthomclFilterFasta.pl” (Chen et al. 2006). The “orthomclFilterFasta.pl” script filters away poor-quality sequences based on their length and percent stop codons. Default parameters were used, which retains only sequences with a minimum length of 20 characters, and fewer than 10% stop codons. This step resulted in the exclusion of 29 out of 894,528 input sequences (0.0032%). An all-versus-all NCBI BLAST v2.7.1 was then used with default parameters, in order to find the similarity score between sequences. Matches with an *E*-value <10^−6^ were retained. Orthologs, in-paralogs, and co-orthologs were then identified using the OrthoMCL script “OrthomclPairs.pl” (Chen et al. 2006) before clustering using MCL. An MCL inflation parameter of 2.2 was used in order to identify clusters. Each group was required to have at most one species containing multiple sequences. When more than one sequence from a single species was present, the longest sequence was selected to remain in the group, with the others removed.

Putative orthologs were inferred using OrthoFinder v2.2.7, used in conjunction with BLAST, with default parameters. When applying the same criteria as for OrthoMCL for generating single copy orthologs (i.e., at most sequence per species), no orthologous groups were recovered. As a workaround, within each orthologous group, we removed all sequences from species that appeared multiple times.

#### Phylogenetic inference

Each orthologous group that contained a minimum of 15 protein sequences, of the 30 total, representing unique species were aligned using MUSCLE (Edgar 2004), using default parameters. All spurious sequences, and poorly aligned regions of the multiple sequence alignments, were then removed using trimAl (Capella-Gutiérrez et al. 2009), using the -automated1 flag. Supermatrices were then constructed by concatenating all of the remaining alignments, with missing sequences treated as gaps. The final alignment was subsequently reduced to only contain sites in which more than 60% were occupied by amino acids.

Species trees were constructed using IQ-TREE, with 1000 ultrafast bootstrap replicates (Hoang et al. 2018). Model selection was determined by ModelFinder, with a gamma rate of heterogeneity, which found the best fitting model for each supermatrix (Table 2). We also computed IQ-TREE trees using the C20 mixture model (site-specific frequency model) (Wang et al. 2018), but the support values were low across all methods (Supplemental Fig. S3), and thus we decided against using them further in our analyses. In addition to the maximum likelihood trees, we constructed Bayesian trees using PhyloBayes MPI v1.5a, using the CAT + GTR + G4 model. Convergence information is provided in Table 3.

**Table 2.**
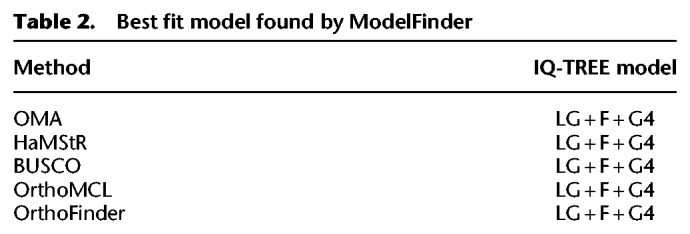
Best fit model found by ModelFinder

**Table 3.**
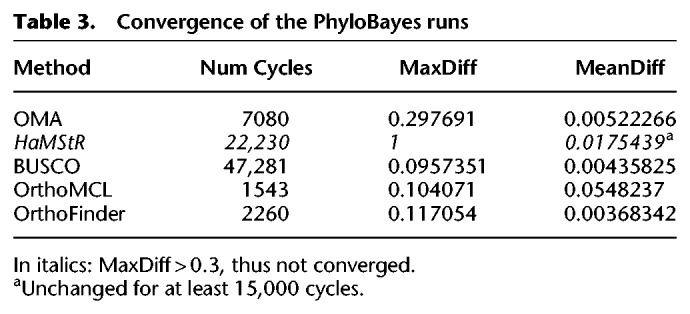
Convergence of the PhyloBayes runs

#### Group subsampling analyses

As the number of orthologous groups can depend on the parameters for each of the inference methods, we subsampled the data so that the supermatrices were of equivalent size. This allows us to assess the quality of each of the groups. For each orthology method, from the set of predicted orthologous groups with at least 50% of the species, a number of groups were selected at random, without repeats, but ensuring that every species was represented in at least one group. The groups were concatenated in order to construct supermatrices using the same process mentioned previously, when constructing full species trees. Species trees were then constructed using IQ-TREE, with a WAG + I model of evolution. WAG + I was chosen because, after preliminary tests on a selection of trees, it was found to give good trees in a relatively short amount of time. This process was repeated 50 times for each orthology inference method. The number of orthologous groups were 50, 100, 200, and every further 200 up to 2000. When the number of orthologous groups to select exceeded the total number of orthologous groups a method inferred (i.e., over 400 groups for BUSCO and over 600 groups for OrthoMCL, etc.), no further supermatrices could be constructed.

**Figure 6.**
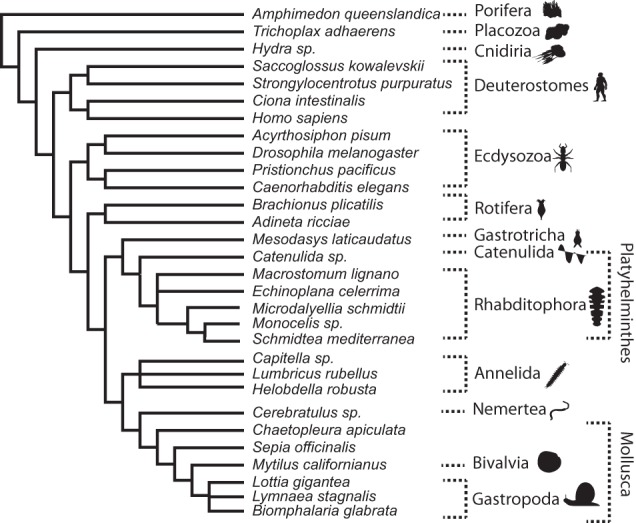
Model tree based on the literature (see Methods).

The Robinson-Foulds distances between the model tree (Fig. 6) and each of the species trees were computed. In order to account for polytomies, the upper bound for the Robinson-Foulds distance was calculated. This is achieved by counting each missing split as a contribution to the Robinson-Foulds score, assuming that each missing split resulted in a conflicting topology. The distance was normalized by dividing by the maximum possible Robinson-Foulds score (2·(*n*–3), where *n* is the number of taxa).

### Software availability

To facilitate reproducibility, we are providing custom Python script as Supplemental Code. Intermediate and output data of the Lophotrochozoan phylogenomic analysis are provided as Supplemental Data.

## Supplementary Material

Supplemental Material
