# Supplementary material for: OMA standalone: orthology inference among public and custom genomes and transcriptomes
Source: Genome Res. 2019 Jul;29(7):1152–63. doi: 10.1101/gr.243212.118 (PMC6633268; doi:10.1101/gr.243212.118)
Supplement: Supplemental Material [file supp_29_7_1152__index.html]

OMA standalone: orthology inference among public and custom genomes and transcriptomes — Supplemental Material 

# OMA standalone: orthology inference among public and custom genomes and transcriptomes

## Supplemental Material

- Supplemental\_Table\_S1.docx
- Supplemental\_Code.zip
- Supplemental\_Data.zip
- Supplemental\_Figures\_.pdf
