# Supplementary material for: OMA standalone: orthology inference among public and custom genomes and transcriptomes
Source: Genome Res. 2019 Jul;29(7):1152–63. doi: 10.1101/gr.243212.118 (PMC6633268; doi:10.1101/gr.243212.118)
Supplement: Supplemental Material [file supp_gr.243212.118_Supplemental_Table_S1.docx]

**Supplemental Table S1: Summary of support for major clades in trees obtained using the different methods.** P indicates presence of a clade in PhyloBayes trees (Figure 4). L indicates presence of a clade in maximum likelihood tree, constructed using IQ-TREE with the model chosen by ModelFinder (Supplementary Figure 2). Boldface indicates branch support >=0.95.

| **Hypothesis** | **OMA** | **OrthoMCL** | **HaMStR*** | **BUSCO** | **OrthoFinder** |
| --- | --- | --- | --- | --- | --- |
| Monophyly of Lophotrochozoa [(Dunn et al. 2008; Kocot et al. 2017; Telford et al. 2015)](https://paperpile.com/c/tJN5XX/MGy2+HIKt+pphi) | P  - | **P**  - | **P**  **L** | P  - | **P**  L |
| Gastropoda sister to Bivalvia [(Kocot et al. 2011)](https://paperpile.com/c/tJN5XX/3bJqo) | **P**  **L** | -  - | -  - | -  - | P  L |
| Annelida sister to (Mollusca + Nemertea)  [(Egger et al. 2015)](https://paperpile.com/c/tJN5XX/AVP1) | **P**  **L** | **P**  **L** | -  **L** | -  **L** | -  **L** |
| Monophyly of Deuterostomes | **P**  **L** | -  L | **P**  **L** | **P**  **L** | **P**  **L** |
| Rotifera sister to rest of Lophotrochozoa  [(Laumer et al. 2015; Marlétaz et al. 2019)](https://paperpile.com/c/tJN5XX/P0lH+k5Tq) | **P**  - | -  - | **P**  - | **P**  - | **P**  - |
| Catenulida sister to Rhabditophora  [(Egger et al. 2015)](https://paperpile.com/c/tJN5XX/AVP1) | **P**  **L** | **P**  L | **P**  **L** | **P**  **L** | **P**  **L** |
| Microdalyella sister to (Monocelis + Schmidtea)  [(Egger et al. 2015; Laumer et al. 2015)](https://paperpile.com/c/tJN5XX/AVP1+P0lH) | **P**  L | **P**  **L** | P  L | P  L | P  L |
| Monophyly of Annelida | **P**  **L** | **P**  **L** | **P**  **L** | **P**  **L** | **P**  **L** |
| Sister Clade of Gastrotricha to Platyhelminthes  [(Egger et al. 2015; Laumer et al. 2015)](https://paperpile.com/c/tJN5XX/AVP1+P0lH) | **P**  L | **P**  - | **P**  **L** | **P**  **L** | **P**  - |
| Cephalopods are outgroup to Gastropods and Bivalvia  [(Kocot et al. 2011)](https://paperpile.com/c/tJN5XX/3bJqo) | **P**  L | -  - | -  - | -  L | **P**  L |
| Polyplacophora are outgroup to Cephalopods [(Kocot et al. 2011)](https://paperpile.com/c/tJN5XX/3bJqo) | **P**  **L** | -  - | -  - | -  L | **P**  L |
| **Total majority outcomes** | 11 PhyloBayes  9 IQ-TREE | 6 PhyloBayes  5 IQ-TREE | 7 PhyloBayes  7 IQ-TREE | 7 PhyloBayes  8 IQ-TREE | 10 PhyloBayes  9 IQ-TREE |

*The PhyloBayes tree computed from HaMSTR data did not converge after 900,000 CPU hours.
