# Supplementary material for: OMA standalone: orthology inference among public and custom genomes and transcriptomes
Source: Genome Res. 2019 Jul;29(7):1152–63. doi: 10.1101/gr.243212.118 (PMC6633268; doi:10.1101/gr.243212.118)
Supplement: Supplemental Material [file supp_gr.243212.118_Supplemental_Figures_.pdf]

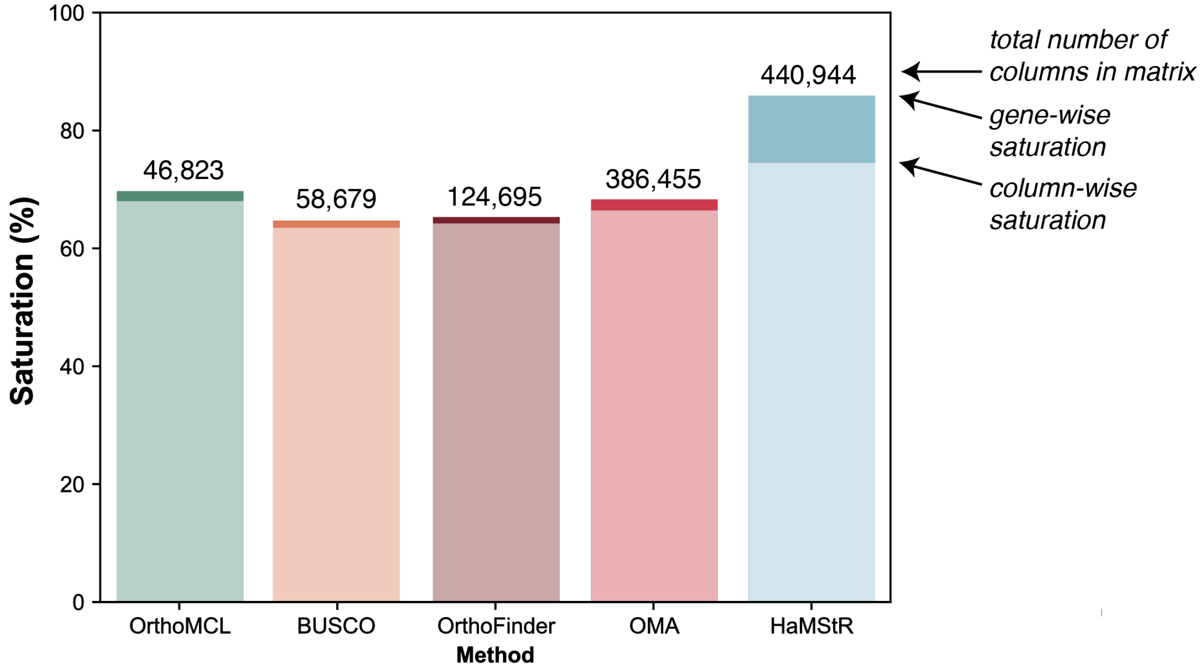

Supplemental Figure S1: Data matrix saturation (occupancy), in terms of average proportion of species represented in each group computed using MARE (Meyer and Misof 2011), average proportion of characters per site (pastel). The total number of characters is numbers above each bar, with the methods ordered accordingly.

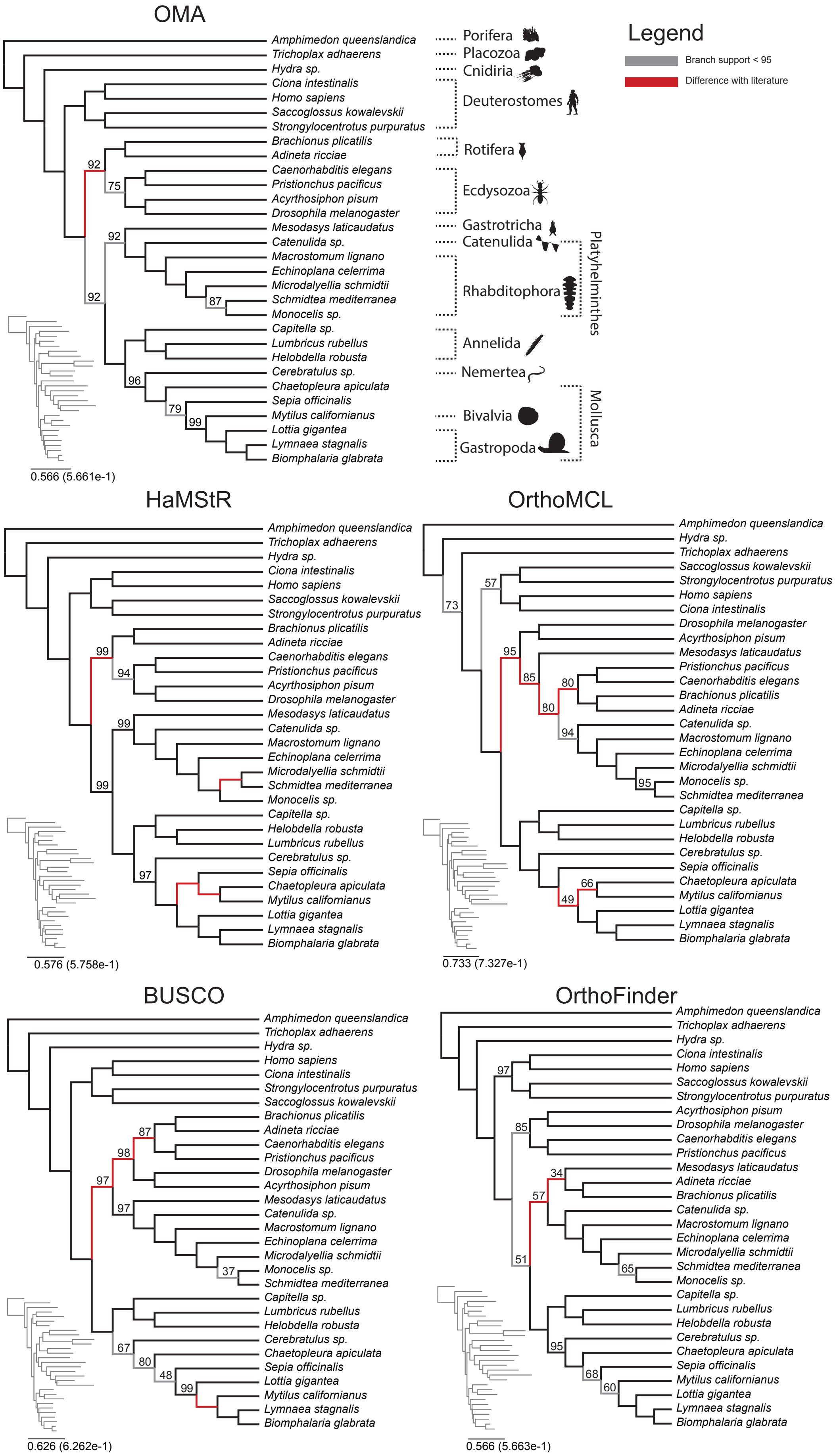

Supplemental Figure S2: Comparison of trees obtained using IQ-TREE with model selection for different datasets. Branches which are at odds with the literature are in red; else they are displayed in grey (branch support < 0.95) or in black. Only branch support measures below 1 are displayed.

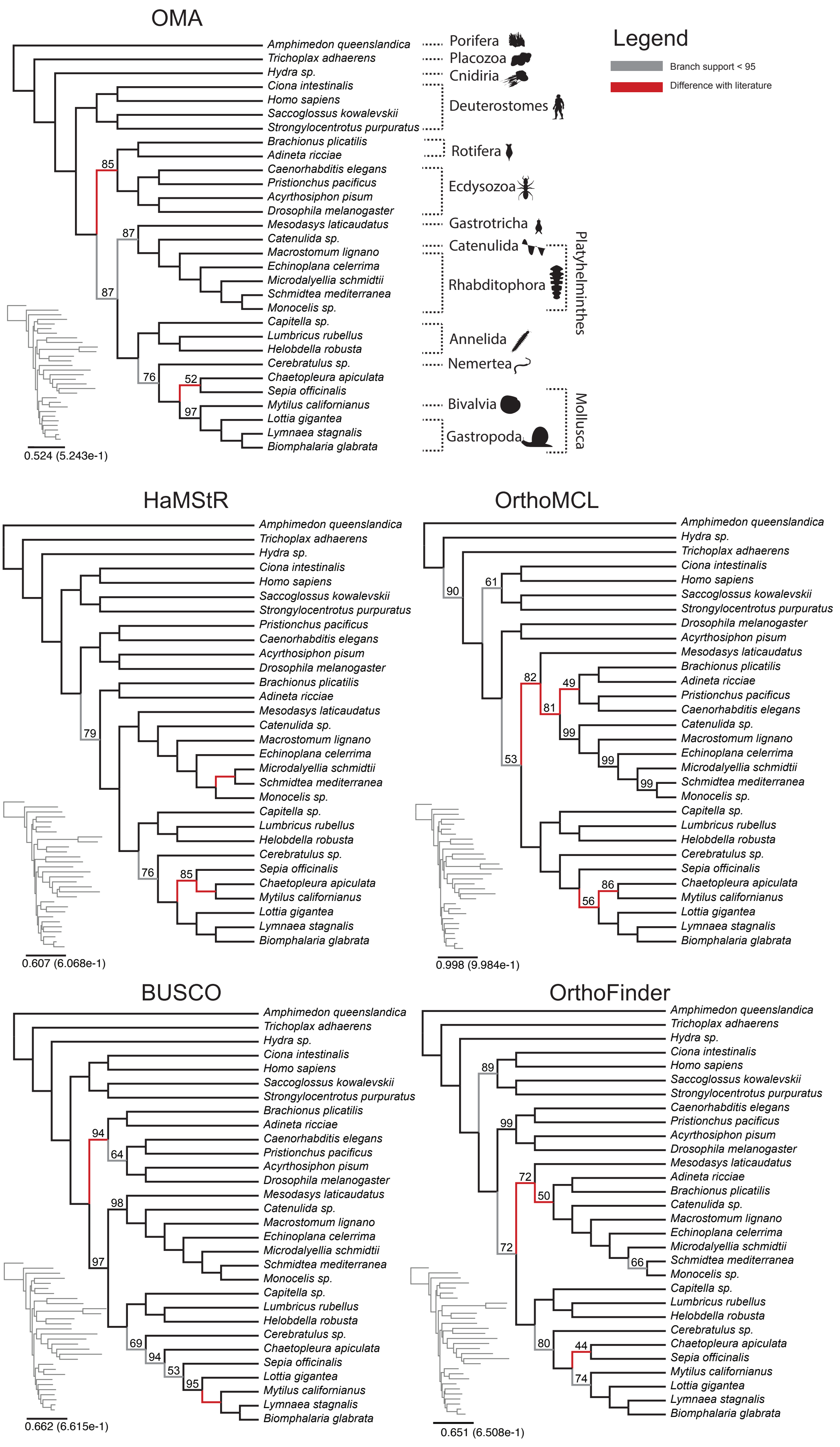

Supplemental Figure S3: Comparison of trees obtained using IQ-TREE C20 (site-specific frequency models) for different datasets. Branches which are at odds with the literature are in red; else they are displayed in grey (branch support < 0.95) or in black. Only branch support measures below 1 are displayed. Because the support measures were quite low throughout, we did not use these trees to compare methods.
